# Comparing lesion detection efficacy and image quality across different PET system generations to optimize the iodine-124 PET protocol for recurrent thyroid cancer

**DOI:** 10.1186/s40658-021-00361-y

**Published:** 2021-02-15

**Authors:** David Kersting, Walter Jentzen, Miriam Sraieb, Pedro Fragoso Costa, Maurizio Conti, Lale Umutlu, Gerald Antoch, Michael Nader, Ken Herrmann, Wolfgang Peter Fendler, Christoph Rischpler, Manuel Weber

**Affiliations:** 1 Department of Nuclear Medicine, University Hospital Essen, University of Duisburg-Essen, Hufelandstrasse 55, 45147 Essen, Germany; 2 West German Cancer Center (WTZ), Essen, Germany; 3 German Cancer Consortium (DKTK), Essen and Dusseldorf, Germany; 4 Siemens Medical Solutions USA, Inc., Knoxville, TN USA; 5 Department of Diagnostic and Interventional Radiology and Neuroradiology, University Hospital Essen, 45147 Essen, Germany; 6 Medical Faculty, Department of Diagnostic and Interventional Radiology, University Dusseldorf, 40225 Dusseldorf, Germany

**Keywords:** Iodine-124 PET, Digital PET/CT, Biograph Vision, Detectability, Differentiated thyroid cancer

## Abstract

**Background:**

In recurrent differentiated thyroid cancer patients, detectability in ^124^I PET is limited for lesions with low radioiodine uptake. We assess the improvements in lesion detectability and image quality between three generations of PET scanners with different detector technologies. The results are used to suggest an optimized protocol.

**Methods:**

Datasets of 10 patients with low increasing thyroglobulin or thyroglobulin antibody levels after total thyroidectomy and radioiodine therapies were included. PET data were acquired and reconstructed on a Biograph mCT PET/CT (whole-body, 4-min acquisition time per bed position; OSEM, OSEM-TOF, OSEM-TOF+PSF), a non-TOF Biograph mMR PET/MR (neck region, 4 min and 20 min; OSEM), and a new generation Biograph Vision PET/CT (whole-body, 4 min; OSEM, OSEM-TOF, OSEM-TOF+PSF). The 20-min image on the mMR was used as reference to calculate the detection efficacy in the neck region. Image quality was rated on a 5-point scale.

**Results:**

All detected lesions were in the neck region. Detection efficacy was 8/9 (Vision OSEM-TOF and OSEM-TOF+PSF), 4/9 (Vision OSEM), 3/9 (mMR OSEM and mCT OSEM-TOF+PSF), and 2/9 (mCT OSEM and OSEM-TOF). Median image quality was 4 (Vision OSEM-TOF and OSEM-TOF+PSF), 3 (Vision OSEM, mCT OSEM-TOF+PSF, and mMR OSEM 20-min), 2 (mCT OSEM-TOF), 1.5 (mCT OSEM), and 1 (mMR OSEM 4 min).

**Conclusion:**

At a clinical standard acquisition time of 4 min per bed position, the new generation Biograph Vision using a TOF-based image reconstruction demonstrated the highest detectability and image quality and should, if available, be preferably used for imaging of low-uptake lesions. A prolonged acquisition time for the mostly affected neck region can be useful.

**Supplementary Information:**

The online version contains supplementary material available at 10.1186/s40658-021-00361-y.

## Background

Elevated thyroglobulin (Tg) levels in differentiated thyroid cancer (DTC) patients after total thyroidectomy and radioiodine therapies are associated with detectable recurrence [1] and poor outcome [2, 3]. According to the current American Thyroid Association (ATA) guideline [4], DTC patients with elevated Tg levels undergo a diagnostic iodine-131 (^131^I) whole-body scan (~ 185 MBq). Radioiodine therapy (1.85–7.40 GBq) is performed if radioiodine-avid lesions are identified. However, diagnostic whole-body scans are limited, e.g., by a low diagnostic accuracy of 36% in biochemical recurrent intermediate- or high-risk DTC patients [5]. A suitable alternative is iodine-124 (^124^I) positron emission tomography (PET) imaging. For instance, studies demonstrated that 50% more foci of radioiodine-avid lesion compared to diagnostic whole-body scans were identified [6] and a high level of agreement (95%) between ^124^I PET and intra-therapeutic ^131^I single photon emission computed tomography/computed tomography (SPECT/CT) was found [7]. However, false negative results may arise for lesions with ^124^I activities below the PET scanner’s size-dependent minimum detectable activity [7–9]. The recently introduced “digital” silicon photomultiplier-based (SiPM-based) PET/CT systems show a higher coincidence time resolution and a higher spatial resolution [10] (compared to conventioanl PET/CT systems). These properties were associated with a higher image quality and a higher detectability of small lesions in phantom settings and clinical applications in different studies using 2-deoxy-2-[fluorine-18]-fluoro-D-glucose (^18^F-FDG) [11–14] and, most recently, using [gallium-68]gallium-prostate-specific membrane antigen-11 [15].

In DTC patients with low but increasing Tg levels, the recurring lesions are often small and exhibit low ^124^I uptake values. Moreover, ^124^I possesses a low positron branching ratio (23%) and ^124^I PET emission data are contaminated by a high prompt gamma fraction (about one-third of total coincidences) requiring advanced image correction prior to image reconstruction [16]. In combination with typically low administered ^124^I activities, these properties result in low count statistics and noisy images — one reason why ^124^I PET especially benefits from time-of-flight (TOF) image reconstructions [17]. Therefore, the improved TOF performance and sensitivity of SiPM-based PET/CT systems might have a particularly pronounced effect for ^124^I in this patient cohort. In addition, a combination of TOF and point spread function modeling (PSF) image reconstruction bears potential for further quality improvement [18]. In a comparison using ^18^F-FDG on a digital PET/CT system, OSEM-TOF+PSF reached improvements to OSEM-TOF image reconstruction in image quality, image sharpness, and lesion conspicuity [19].

We hypothesize that the use of SiPM-based PET systems will lead to relevant improvements in thyroid cancer detectability and image quality. In this study, datasets of 10 DTC patients were evaluated, who received ^124^I imaging on three PET systems with different detector technologies. We aim to assess the influence of SiPM-based PET on detection efficacy for lesions in the neck region, and on visual image quality. Furthermore, the data are used to suggest an ^124^I PET protocol for recurrent thyroid cancer considering, inter alia, the acquisition time duration, and the amount of applied ^124^I activity.

## Methods

### PET scanners

All patients were scanned on two PET/CT systems, a SiPM-based Biograph Vision 600 and a photomultiplier tube (PMT)-based Biograph mCT, and one PET/MR (magnetic resonance imaging) system, an avalanche photodiode (APD)-based Biograph mMR (all from Siemens Healthineers, Erlangen, Germany). A short description of the scanner specifications is shown as Supplemental Material.

### Patient characteristics

The local institutional ethics committee (University of Duisburg-Essen) approved the study (Ethics protocol number 20-9203-BO). In the following, all scaled variables are presented as mean ± standard deviation (SD), and ordinal data are presented as median (interquartile range = IQR).

We routinely perform whole-body ^124^I PET/CT on the analog Biograph mCT as well as a prolonged scan duration PET/MR of the neck region using the Biograph mMR in DTC patients with low increasing Tg and/or Tg antibody levels after total thyroid ablation. Since its introduction at our center, patients of this rare group [20] were additionally examined on the digital Biograph Vision PET/CT system, resulting in 10 DTC patients examined on three PET systems until April 2020. These patients were retrospectively evaluated in this study. Specifically, datasets of these 5 males and 5 females after total thyroidectomy and adjuvant radioiodine therapies were included (7 with papillary and 3 with follicular thyroid cancer, mean ± SD age 52 ± 18 years). In 9 patients, Tg levels were elevated in the low measurable range (mean unstimulated Tg value of 1.8 ± 1.8 ng/mL, range 0.1–5.5 ng/mL), in one patient Tg antibody levels were elevated (273 IU/mL) with non-measurable Tg. Serum thyroid stimulating hormone level stimulation (≥ 30 mU/L) was achieved by levothyroxine withdrawal or intramuscular recombinant human thyroid stimulating hormone injection prior to ^124^I application. Detailed patient characteristics are given in the Supplemental Material.

### Acquisition and image reconstruction

The initial PET scans were acquired 17.1 ± 1.0 h after oral application of 38.3 ± 2.1 MBq of ^124^I; acquisitions on all three scanners were performed within an interval of 4.7 ± 2.9 h (details in Supplemental Material). For the Vision and mCT, the examinations included whole-body PET/CT scans from head to thigh using 5–8 bed positions; the acquisition time duration was 4 min per bed position. For the mMR, a neck scan (a single bed position) was acquired at 20-min acquisition time in list-mode (allowing for resampling of 4-min acquisition time data). PET/CT scans started with a whole-body spiral CT in low-dose technique (tube voltage of 120 kVp, tube current time product of 15 mAs, beam pitch of 1.0, and slice width of 5 mm) without application of contrast agent. Subsequently, the PET scan was acquired. On the PET/MR, simultaneous with PET, T1-weighted MR images were acquired using a VIBE sequence after application of gadolinium-based contrast agent.

All scanners allow for iterative image reconstruction algorithms. Image reconstructions were performed using (three-dimensional) ordinary Poisson ordered-subsets expectation maximization (OSEM), with TOF reconstruction alone (OSEM-TOF), or with both TOF and PSF (OSEM-TOF+PSF). On the mMR, the slow timing characteristics of avalanche photodiodes preclude the TOF reconstruction. All PET data were reconstructed using our clinically standard reconstruction protocols that were optimized for quantitative ^124^I imaging [21] and are listed in Table 1. They were corrected for scatter, randoms, attenuation, dead time, decay, and normalization. For PET/MR images, attenuation correction was based on an attenuation map (μ-map) derived from a 3-dimensional Dixon-VIBE MR sequence. In addition, for all PET systems, the same prompt gamma coincidence correction method is by default implemented in the PET reconstruction algorithm for radionuclides emitting prompt gammas such as ^124^I [22, 23].

**Table 1:** Standard clinical reconstruction parameters for the different PET scanners

Iterative reconstruction	Scanner type	Iterations × subsets	Gauss filter (mm)	Voxel size (mm^**3**^)
OSEM	Vision	10 **×** 5	4	1.7 **×** 1.7 **×** 2.0
	mCT	3 **×** 24	3	2.0 **×** 2.0 **×** 2.0
	mMR	3 **×** 21	4	2.1 **×** 2.1 **×** 2.0
OSEM-TOF	Vision	4 **×** 5	4	1.7 **×** 1.7 **×** 2.0
	mCT	2 **×** 21	3	2.0 **×** 2.0 **×** 2.0
	mMR	–	–	–
OSEM-TOF+PSF	Vision	4 **×** 5	4	1.7 **×** 1.7 **×** 2.0
	mCT	2 **×** 21	3	2.0 **×** 2.0 **×** 2.0
	mMR	–	–	–

### Image analysis

#### Detection efficacy and visual image quality

All PET datasets were assigned a random number as identifier. The images were anonymized and interpreted in random order in a consensus read by three nuclear medicine residents. The readers were blinded to clinical information, PET scanner type, and acquisition protocol to exclude prior knowledge from previously evaluated studies about the localizations of lesions. Focal ^124^I uptake was reported in five separate anatomical regions: local thyroid bed, cervical lymph nodes, extra-cervical lymph nodes (only PET/CT), lungs (only PET/CT), and bones. For each lesion, the maximum standardized uptake value (SUV_max_), the maximum activity concentration (AC_max_), and the local signal-to-background ratio (ratio of lesion SUV_max_ to background SUV_bgr_ derived from a region of interest surrounding the lesion) were determined. The long-axis diameters of the lesions were measured on the MR images; for morphologically not clearly definable lesions, an upper size limit was estimated from the PET data using an iterative volume segmentation approach [24]. These functional and morphological properties were assessed to correlate their values with differences in detectability. Moreover, the image quality was visually assessed in transversal slices of the PET images on an established 5-point Likert-like scale from 1 (poor) to 5 (excellent) [25].

#### Lesion- and patient-based analysis

For the lesion-based analysis, the total number of ^124^I-positive lesions in the neck region were counted across all PET systems, acquisition time durations, and image reconstruction algorithms. A total of nine lesions were detected in the neck region; the 20-min acquisition time reference scan on the mMR was the only acquisition in which all lesions were detectable, no additional lesions were reported on the whole-body PET/CT images. The detectability in PET images is dependent on the acquisition time [26]. We therefore used PET data acquired for a clinical standard acquisition time of 4 min per bed position to compare the detectability across the different generations of PET systems. Detection efficacy was defined as the number of lesions identified divided by nine lesions (in the reference image). For the patient-based analysis, the total number of radioiodine-positive patients was counted, and the distinct levels of agreement were determined.

### Statistical analysis

Statistical analyses were performed using OriginPro 2020b (OriginLab, Northampton, USA). For assessing changes in visual image quality (ordinal data) a Mann-Whitney test was used. A *p* value < 0.05 was considered statistically significant.

## Results

### Lesion-based and patient-based detection efficacy analysis

**Fig. 1:**
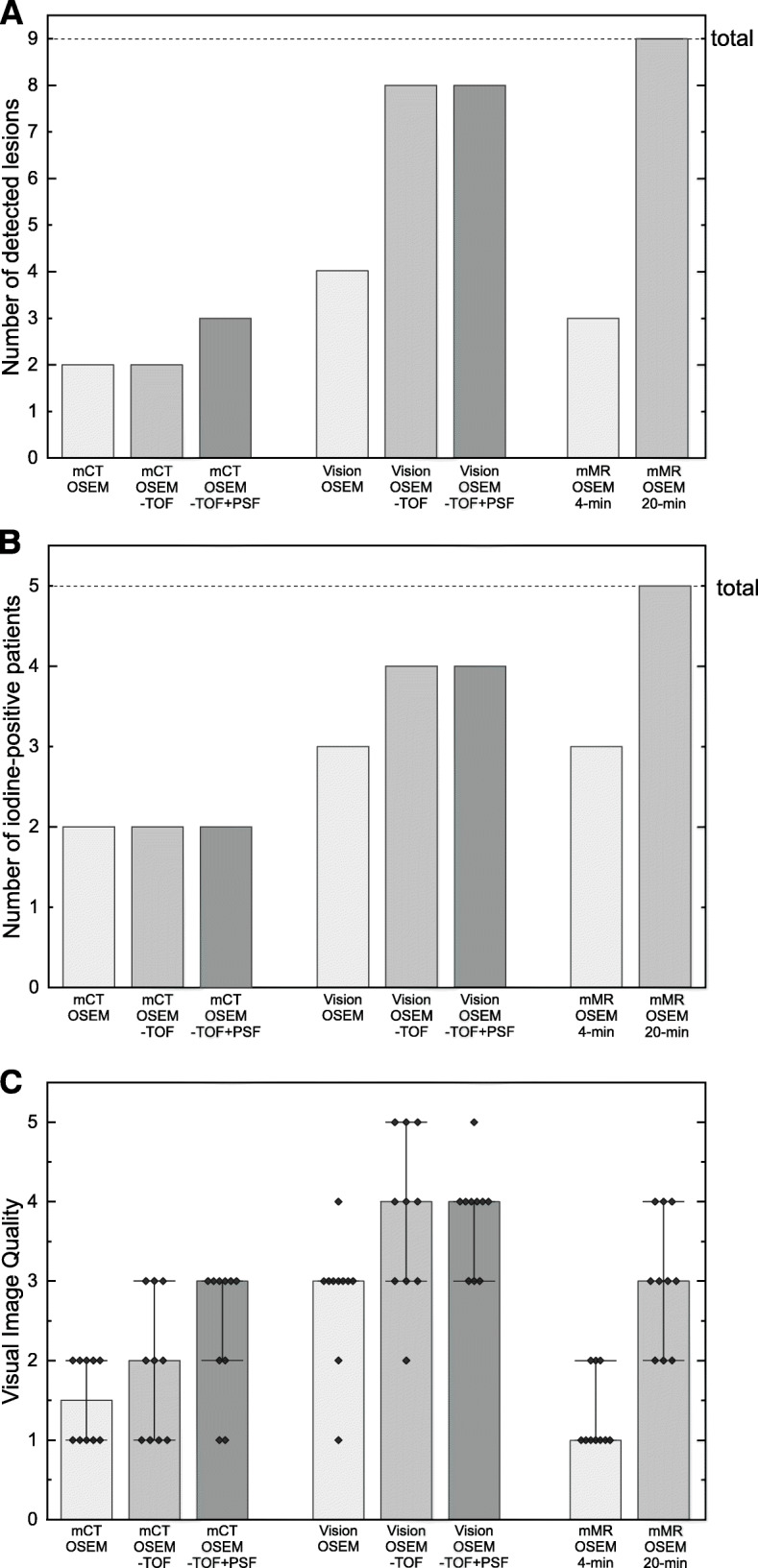
Bar charts of **a** lesion-based detection efficacy, **b** patient-based detection efficacy, and **c** visual image quality (diamonds: data points, bar: median, whiskers: quartiles). The detection efficacy was evaluated using as reference the five-fold prolonged scan duration image acquired on the PET/MR system

Detection efficacy results are summarized in Fig. 1a, b and Table 2, detailed lesion characteristics are presented in Table 3. If not otherwise stated, the individual values presented in the subsequent paragraphs were taken from images acquired on the Vision and reconstructed using OSEM-TOF.

**Table 2:** Lesion-based detection efficacy analysis

Lesion ID	Patient ID	mCT OSEM	mCT OSEM-TOF	mCT OSEM-TOF+PSF	Vision OSEM	Vision OSEM-TOF	Vision OSEM-TOF+PSF	mMR 4-min	mMR 20-min
1	1	1	1	1	1	1	1	1	1
2	1	0	0	1	1	1	1	0	1
3	1	0	0	0	0	1	1	0	1
4	2	0	0	0	0	0	0	0	1
5	3	0	0	0	0	1	1	0	1
6	5	1	1	1	1	1	1	1	1
7	5	0	0	0	0	1	1	0	1
8	9	0	0	0	1	1	1	1	1
9	9	0	0	0	0	1	1	0	1

Detection is indicated by “1”, while missed detection is indicated by “0”

**Table 3:** Lesion characteristics

Lesion ID	AR	Size (mm)	SUV_**max**_ mMR 20-min	SUV_**max**_ Vision OSEM-TOF	AC_**max**_ mMR 20-min (kBq/mL)	AC_**max**_ Vision OSEM-TOF (kBq/mL)	SBR mMR 20-min
1	CLN	13	15.2	14.0	4,6	4.2	42.6
2	CLN	17	8.8	9.9	2.6	3.5	25.7
3	CLN	9	3.1	3.4	0.9	1.0	10.7
4	CLN	7	2.3	–	0.8	–	12.6
5	Bone	< 6	8.4^a^	3.1	3.2^a^	1.2	14.4
6	TB	16	10.2	9.9	4.7	4.6	29.6
7	CLN	6	3.0	2.2	1.4	1.0	8.4
8	TB	<6	5.6	5.1	2.4	2.8	7.1
9	CLN	4	3.0	1.8	1.3	0.9	6.5

*AR* Anatomical region, *CLN* Cervical lymph node, *TB* Thyroid bed, *SBR* Signal-to-background ratio (only reported for the 20-min acquisition on the mMR, in which all lesions were detectable)

^a^Potentially artificially elevated activity concentration in PET/MR attenuation correction, as the bone lesion was osteolytic

**Fig. 2:**
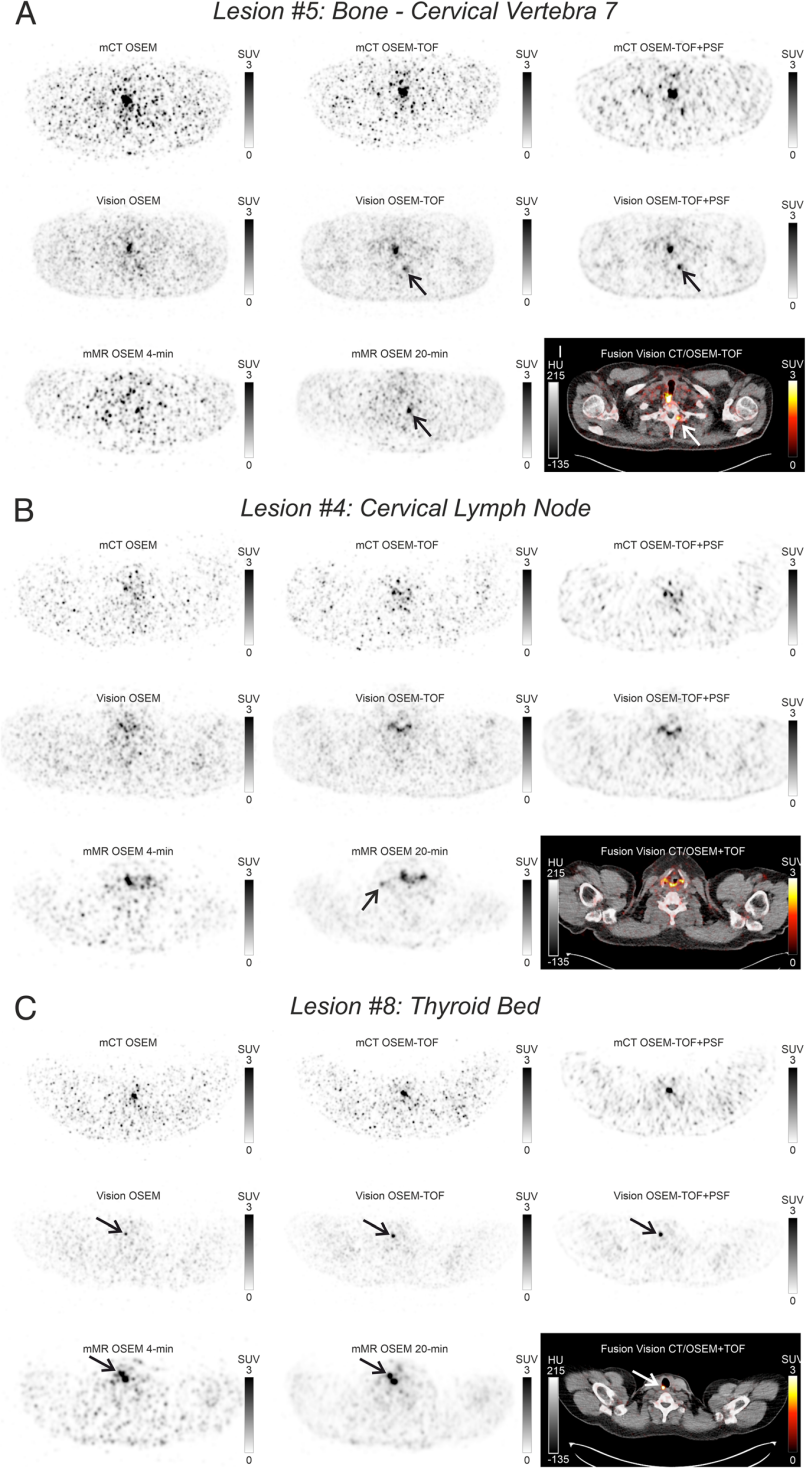
PET(/CT) images of **a** an osteolytic lesion in cervical vertebra 7 (lesion #5), of **b** a cervical lymph node (lesion #4), and of **c** a thyroid bed lesion (lesion #8). Detected lesions are indicated by arrows. In **a** and **c** oesophageal tracer uptake is not visible on all images due to different imaging times

Detection efficacy was 8/9 on the Vision in both OSEM-TOF and OSEM-TOF+PSF images. The only lesion (#4, about 7 mm in diameter), which was not detected (Fig. 2b), had the lowest AC_max_ of 0.8 kBq/mL and SUV_max_ of 2.3 (all values for this specific lesion were measured on the mMR images at 20-min acquisition time). A detection efficacy of 4/9 (lesions #1, #2, #6, #8) was reached on the Vision using OSEM. The four lesions that were only detected in OSEM-TOF and OSEM-TOF+PSF images were small (≤ 9 mm) and of very low AC_max_ (≤ 1.2 kBq/mL) and SUV_max_ (≤ 3.4) (e.g., lesion #5 in Fig. 2a).

Detection efficacy was 3/9 (lesions #1, #6, #8) on the mMR at 4-min acquisition time. The six lesions that were not detected at 4-min acquisition time comprised the five lesions that were not detected using OSEM on the Vision and one cervical lymph node (#2, 17 mm, AC_max_ 3.5 kBq/mL, SUV_max_ 9.9) that was probably not detected, as it was hardly distinguishable from a different lymph node localized directly next to it.

Detection efficacy was 3/9 on the mCT using OSEM-TOF+PSF (lesions #1, #2, #6) and 2/9 (lesions #1, #6) using OSEM or OSEM-TOF. The six lesions that were not detected on the mCT were of small size (≤ 9 mm), low AC_max_ of ≤ 2.8 kBq/mL, and SUV_max_ of ≤ 5.1 (examples in Fig. 2a–c). Figure 2c illustrates the only lesion (#8, < 6 mm, AC_max_ 2.8 kBq/mL, SUV_max_ 5.1) that was detectable on the Vision using OSEM but not on the mCT. The lymph node that was hardly distinguishable (#2, detailed description above) was only detected using OSEM-TOF+PSF. However, OSEM-TOF+PSF induced two additional foci (hilar lymph node and bone, not shown) with probably artificially elevated uptake. These lesions were not discernible in the images from the scanners with higher sensitivity and a critical re-evaluation revealed that the reported uptakes were of the same magnitude as other spots in the background noise of the evaluated images; the hilar lymph node was not included in the scan area of the PET/MR.

As illustrated in Fig. 1b, the patient-based detection efficacy revealed that 5 of the 10 included patients were radioiodine-positive (in the 20-min acquisition time reference scan on the mMR). On the Vision, 4/5 patients were identified using OSEM-TOF or OSEM-TOF+PSF and 3/5 using OSEM. On the mMR, 3/5 patients were identified at 4-min acquisition time. On the mCT, 2/5 patients were identified independent of the image reconstruction algorithm.

### Visual image quality

For the PET/CT systems, the median (IQR) visual image quality showed statistically significant increases from the mCT to the Vision from 1.5 (2–1) to 3 (3–2.75) for OSEM (*p* < 0.005), from 2 (3–1) to 4 (5–3) for OSEM-TOF (*p* < 0.005), and from 3 (3–1.75) to 4 (4–3) for OSEM-TOF+PSF images (*p* < 0.005), respectively (Fig. 1c). For the Vision, the changes from OSEM to OSEM-TOF images (*p* < 0.05) and from OSEM to OSEM-TOF+PSF images (*p* < 0.01) were statistically significant, whereas for the mCT, only the change from OSEM to OSEM-TOF+PSF images was statistically significant (*p* < 0.05).

The non-TOF mMR reached a visual image quality of 1 (2–1) at 4-min acquisition time duration. It was not significantly different from the mCT except for OSEM-TOF+PSF images (*p* < 0.01), but significantly lower than all image reconstructions on the Vision (all *p* < 0.001). When prolonging the acquisition time duration to 20 min, the visual image quality increased to 3 (4–2), a value significantly larger than OSEM (*p* < 0.001) and OSEM-TOF images (*p* < 0.05) on the mCT. Of note, the difference to OSEM-TOF and OSEM-TOF+PSF reconstructions on the Vision did not reach statistical significance.

## Discussion

**Fig. 3:**
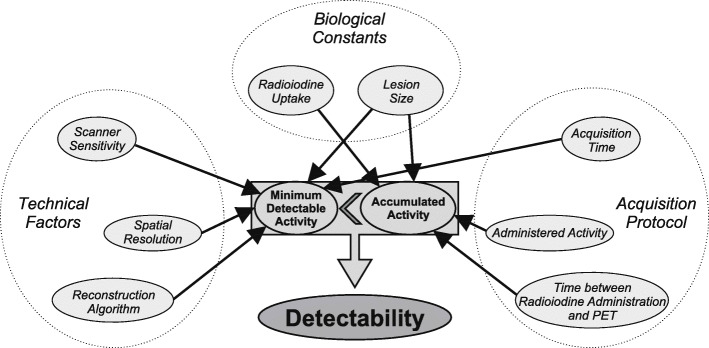
Overview of several factors influencing the lesion detectability in ^124^I PET imaging

A radioiodine-avid DTC lesion is detectable in ^124^I PET/CT if its accumulated activity is above the PET scanner’s size-dependent minimum detectable activity. Several influencing factors in lesion detection shown in Fig. 3 will be discussed to achieve an optimized scan protocol with regards to detection of recurrent DTC lesions.

The minimum detectable activity is largely determined by technical aspects such as scanner’s sensitivity, PET spatial resolution, and image reconstruction algorithms [26]. Sensitivity, time and spatial resolutions are improved in the new generation of SiPM-based PET scanners, leading to an increased detectability [12, 27] and image quality [28]. Recent studies demonstrate that an increased detectability of SiPM-based systems can allow for shorter acquisition time durations in ^18^F-FDG PET without impairing the diagnostic accuracy [29–31]. In this study, we evaluated the effects on ^124^I PET imaging.

At standard acquisition time duration, the Vision outperformed both the mCT and the mMR in detection efficacy and visual image quality; these results were almost independent of the image reconstruction algorithms (Fig. 1 and Table 2). Of note, the image quality on the mMR was relatively low compared to its detection efficacy. An explanation may be associated with non-TOF modeling on this PET system leading to an increase in image noise that impairs visual image quality [32]. In OSEM-TOF and OSEM-PSF+TOF images, the Vision reached, in standard acquisitions, comparable results to those in five-fold prolonged acquisitions on the mMR, which we used as reference. As expected, the missed lesions on the old generation systems were small and of low radioiodine uptake (Table 3). We therefore propose, if available, image acquisition on a new generation SiPM-based PET system for recurrent DTC patients with increasing Tg or Tg antibody levels in the low measurable range. The observed differences in detectability across the PET scanners might not hold for patients with only large and/or high radioiodine uptake lesions that might be detectable on each PET scanner.

Regarding the choice of image reconstruction algorithm, on the Vision the refinements obtained by OSEM-TOF were not further enhanced by OSEM-TOF+PSF, while OSEM-TOF+PSF was necessary on the mCT for improvements in both detection efficacy and image quality. On the mCT, OSEM-TOF+PSF induced probably artificial elevated uptake in two patients rated as a cervical lymph node and a bone pseudo-lesion (these lesions were not discernible in the images from the scanners with higher sensitivity and a critical re-evaluation revealed that the reported uptakes were of the same magnitude as other spots in the background noise of the evaluated images). Moreover, quantification artifacts in small lesions by PSF modeling are a known phenomenon [33] and have yet to be investigated in phantom studies for the new generation SiPM-based ^124^I PET. On the Vision, part of the contrast enhancement results from the better intrinsic spatial resolution due to the smaller crystal size. Therefore, we currently propose the usage of OSEM-TOF or OSEM-TOF+PSF reconstruction for ^124^I PET on the Vision.

Apart from improving the minimum detectable activity, the detectability for small structures can be increased by prolonged acquisition time durations [26, 34]. Comparing the neck PET/MR at standard and five-fold prolonged acquisition time durations, the lesion-based detection efficacy was increased by a factor of 3 (Fig. 1a). Only one lesion detectable on the prolonged PET/MR acquisitions was missed on the SiPM-based PET/CT (Fig. 2b). Still, emphasis on scan duration of critical anatomical regions might be beneficial. As DTC lymph node metastases are typically — like every detected lesion in this study — localized in the neck region [4], we propose a selectively prolonged acquisition time duration for the neck region. Especially, PET acquisition in continuous bed motion mode, which can itself lead to an improved detectability compared to the stop-and-shoot acquisition mode due to a more uniform axial sensitivity [35], allows for easy emphasis of particular body regions. The table speed velocity could be decreased for the neck region. Of the evaluated PET systems, only the Vision is capable of scanning in continuous bed motion mode. An analysis of the effects on detectability and image quality would require an additional study.

A further possibility for improving the detectability is to increase the accumulated ^124^I activity in the lesion by higher administered activities. In a previous phantom study [8], an ^124^I activity of 74 MBq was calculated to yield a detectability for small lesions similar to intra-therapeutic SPECT imaging after application of 7.4 GBq of ^131^I. The administration of larger amounts of ^124^I may be limited by thyroid stunning (i.e., missing/reduced uptake of radioiodine-avid lesions in intra-therapeutic ^131^I whole-body scans compared to pre-therapeutic radioiodine imaging). Thyroid stunning is controversially discussed [36] and not sufficiently investigated for ^124^I [37]. In the literature, applications of up to 74 MBq [6] are described. We therefore believe that an activity of 74 MBq of ^124^I can safely be administered without risking thyroid stunning. The administration of higher activities could be possible but should be validated in an experimental setting.

Additionally, the time interval between ^124^I administration and PET scan can influence the detectability. Studies of the lesion kinetics revealed an optimal temporal distance of approximately 8 h [38]. At our department, the realization of this interval is logistically not possible, explaining the choice for our protocol with a PET start of 15–19 hours after radioiodine application. However, if possible, a shorter temporal distance could be beneficial to improve the detectability.

Of importance, one lesion that was additionally detected on the Vision and on the prolonged neck PET/MR scan (lesion #5, osteolytic lesion in cervical vertebra 7, see Fig. 2a) caused a change in patient management, that is, the patient received a radioiodine therapy with an amount of 6.1 GBq of ^131^I. Regarding the other additionally detected lesions, treatment by radioiodine therapy was already determined by other lesions in the same patient or watchful waiting was performed (due to other radioiodine-negative metastases in the same patient or equivocal dignity in case of low uptake in the thyroid bed).

The different types of co-registered morphological images (i.e., CT or MR) might influence clinical patient management [39]. On the one hand, neck PET/MR was described as superior to PET/CT in identifying morphological correlates to focal ^124^I uptake, particularly for small lymph nodes and can increase the diagnostic certainty [39]. On the other hand, PET quantification can be challenging, as attenuation correction by MR data is limited and the neck region comprises different tissue types in close proximity. However, it was reported that ^124^I PET quantification from PET/MR data is reliable and can be used for dosimetry planning prior to radioiodine therapy [40]. We therefore propose to perform an additional PET/MR of the neck region, if available and if tolerated by the patient. Alternatively, an additional MR scan could be performed and co-registered with the PET/CT scan. The influence on the detectability should be evaluated in a clinical study.

There are four main limitations in this study. First, the number of patients and ^124^I-positive lesions is low possibly resulting in a low statistical power. A low power can lead to an overestimation of small effects [41]. Additional phantom measurements could be beneficial to verify the results of this study. Thus, it is not possible to estimate from this study whether the improved detectability and image quality have a relevant impact on patient outcome. However, the number of detected lesions is typically low in this selected patient group and the number of patients with these characteristics is generally small [20]. Second, the temporal distances between application of ^124^I and PET start among the evaluated PET systems differed. However, we do not expect a pronounced effect as all scans were performed within a mean time interval of 4.7 ± 2.9 h (maximum 8.7 h) that is relatively small compared to the effective ^124^I half-lives of the lesions (previously reported in the range of 59–116 h) [42–45]. Third, a PET start at an optimal time of 8 h after ^124^I administration could lead to higher accumulated activities and higher signal-to-background ratios and, thus, reduce the observed benefits of a high-sensitivity PET scanner. Fourth, on the PET/MR system only one bed position was used while on the PET/CT systems PET data were acquired in step and shoot acquisition mode with overlapping bed positions. Thus, on the PET/MR, lesions on the end slices of the reconstructed images might be impaired by a non-uniform axial sensitivity profile.

## Conclusion

In the evaluated case series of 10 DTC patients at clinical standard acquisition time of 4 min per bed position, the use of the new generation SiPM-based PET/CT (Biograph Vision) and OSEM-TOF or OSEM-TOF+PSF image reconstruction resulted in the highest lesion detection efficacy and visual image quality. The Biograph Vision should, if available, be preferred over the PMT-based Biograph mCT and the APD-based Biograph mMR for standard acquisition time PET of DTC patients with potential low-uptake lesions. As the detectability was dependent on the acquisition time, a selectively prolonged scan duration should be implemented for the neck region.

## Supplementary Information

**Additional file 1: Supplemental Table S1.** PET Scanner Specifications. **Supplemental Table S2.** Detailed patient characteristics and PET time intervals. **Supplemental Table S3.** Detailed PET time intervals.

## Data Availability

The datasets generated and/or analysed during the current study are not publicly available due to privacy legislation but are available from the corresponding author on reasonable request.
