# Supplementary material for: Comparing lesion detection efficacy and image quality across different PET system generations to optimize the iodine-124 PET protocol for recurrent thyroid cancer
Source: EJNMMI Phys. 2021 Feb 15;8:14. doi: 10.1186/s40658-021-00361-y (PMC7884562; doi:10.1186/s40658-021-00361-y)
Supplement: Supplementary file 1 — Additional file 1: Supplemental Table S1. PET Scanner Specifications. Supplemental Table S2. Detailed patient characteristics and PET time intervals. Supplemental Table S3. Detailed PET time intervals. [file 40658_2021_361_MOESM1_ESM.pdf]

Supplemental Material to "Comparing Lesion Detection Efficacy and Image Quality across Different PET System Generations to Optimize the Iodine-124 PET Protocol for Recurrent Thyroid Cancer"

SUPPLEMENTAL TABLE S1 PET Scanner Specifications

	Biograph mCT	Biograph Vision	Biograph mMR
Detector material	LSO	LSO	LSO
Detector element dimension (mm$^{3)}$	4x4x20	3.2x3.2x20	4x4x20
Detector elements per block	13x13	16x16	8x8
Total number of detector elements	33,448	60,800	28,672
Signal readout	PMTs (2x2 per block)	SiPM (2x2 per block)	APDs (3x3 per block)
Axial FOV (cm)	21.8	26.3	25.8
Transaxial FOV (cm)	70	78	59
Plane spacing (mm)	2	1.65	2
Image planes	109	119	127
Coincidence time window (ns)	4.1	4.7	5.9
Energy window (keV)	435-650	435-585	430-610
Energy resolution (%)	11	9	14
System time resolution (ps)	540	210	-
NEMA sensitivity (kcps/MBq)	9.7	16.4	15

Supplemental Table S1: Technical specifications for the Biograph mCT, the Biograph Vision and the Biograph mMR. LSO = Lutetium Oxyorthosilicate.

SUPPLEMENTAL TABLE S2: Detailed patient characteristics and PET time intervals

Patient ID	Sex	Age (yrs)	Histo logy	TNM	Tg (ng/mL)	Tg anti-bodies (IU/mL)	No. of RAIT	Cumulated activity of ^{131}I (GBq)	Applied activity of ^{124}I (MBq)	Time of first PET scan after ^{124}I application (h)	Time interval between 1st and 3rd PET scan (h)	Further patient management
1	m	68	PTC	T3N1M1	NM	273	1	3.7	37.9	18.1	2.1	W&W
2	m	59	PTC	T2N1M0	0.12	NM	2	11.3	44.1	16.9	2.4	W&W
3	m	72	FTC	T3N0M1	5.50	NM	1	3.2	38.4	15.1	2.8	RAIT
4	m	32	FTC	T3N0M0	0.41	NM	2	7.4	37.3	17.4	3.3	W&W
5	f	50	PTC	T1bN0M0	1.21	NM	1	6.3	37.3	16.8	3.3	RAIT
6	f	66	PTC	T1N1M0	0.72	NM	1	2.9	36.6	16.4	8.1	W&W
7	m	19	FTC	T3N0M1	2.60	NM	2	9.4	38.5	18.4	7.0	W&W
8	f	57	PTC	T1N1M0	3.50	NM	2	9.0	38.0	18.2	1.9	W&W
9	f	33	PTC	T3N1M0	0.76	NM	1	3.0	37.6	17.0	8.1	RAIT
10	f	63	PTC	T1bN0M0	1.57	NM	1	4.0	36.9	16.8	8.7	W&W

Supplemental Table S2: Tg = Unstimulated Tg. RAIT = Radioiodine therapy. NM = Not measurable. W&W: Watch and wait.

SUPPLEMENTAL TABLE S3: Detailed PET time intervals

Patient ID	Time of PET scan after ^{124}I application on the mCT (h)	Time of PET scan after ^{124}I application on the Vision (h)	Time of PET scan after ^{124}I application on the mMR (h)
1	18.1	19.1	20.2
2	17.9	16.9	19.3
3	15.2	15.9	17.9
4	17.4	18.6	20.7
5	16.8	17.9	20.1
6	16.4	24.5	17.5
7	18.4	25.5	19.7
8	18.2	19.1	20.1
9	17.9	25.2	17.1
10	16.8	25.4	18.2
